# Supplementary material for: Effect of Scapular Fixation on Lateral Movement and Scapular Rotation during Glenohumeral Lateral Distraction Mobilisation
Source: Healthcare (Basel). 2023 Jun 22;11(13):1829. doi: 10.3390/healthcare11131829 (PMC10341311; doi:10.3390/healthcare11131829)
Supplement: Supplementary file 1 [file healthcare-11-01829-s001.zip › healthcare-2413569-supplementary.pdf]

Table S1: Outcomes of ultrasound measurements of caudal movement of humeral head during three magnitudes of GLDM force.

Magnitude of force	$ICC_{3,1}$ (95% CI)	SEM	MDD_{95}
Baseline	0.999 (0.994 – 1.000)	0.01mm	0.04
Low-force GLDM	0.997 (0.987 - 0.999)	0.04mm	0.12
Medium-force GLDM	0.992 (0.961 - 0.998)	0.01mm	0.02
High-force GLDM	0.987 (0.990 - 0.999)	0.00mm	0.01

Abbreviations: ICC3,1: Intraclass Correlation Coefficient, 95%CI: 95% Confidence Level, SEM: Standard Error of Measurement, MDC95: Minimum Detectable Change at the 95% confidence level, GLDM: glenohumeral lateral distraction mobilisation.

Table S2: Outcomes of the magnitude of force applied with GLDM, with and without scapular fixation in lateral movement of the humeral head and scapular movement, by sex.

Variable	Magnitude of GLDM Force	Scapular Fixation	Non-scapular Fixation	Mean difference (95%CI)	Effect Size	p-value
Women						
Lateral movement of the humeral head	Low-force (22.4 ± 4.5N)	1.02 ± 0.64mm	0.96 ± 1.03mm	0.06mm (-0.64 , 0.78) p=0.858	0.07	F=10.670 p=0.001
	Medium-force (63.8 ± 9.0N)	2.30 ± 1.03mm	1.78 ± 1.47mm	0.52mm (-0.22, 1.26) p=0.150	0.41	
	High-force (135.7 ± 14.9N)	3.73 ± 1.73mm	2.53 ± 1.92mm	1.20mm (0.36 , 2.04) p=0.009	0.66	
Scapular movement	Low-force (22.4 ± 4.5N)	55.7 ± 1.0°	57.3 ± 2.0°	-1.7° (-2.83 , -0.47) p=0.011	1.01	F=55.298 p<0.001
	Medium-force (63.8 ± 9.0N)	56.8 ± 1.4°	62.1 ± 4.0°	-5.3° (-7.50 , -3.00) p<0.001	1.78	
	High-force (135.7 ± 14.9N)	58.7 ± 2.7°	70.3 ± 5.6°	-11.6° (-14.75 , -8.51) p<0.001	2.64	
Men						
Lateral movement of the humeral head	Low-force (22.3 ± 3.2N)	1.00 ± 0.69mm	0.63 ± 0.46mm	0.36mm (0.53 , 0.67) p=0.025	0.63	F=19.363 p<0.001
	Medium-force (71.8 ± 8.7N)	2.06 ± 1.25mm	1.50 ± 1.22mm	0.55mm (0.09 , 1.02) p=0.024	0.45	
	High-force (150.1 ± 22.1N)	3.72 ± 2.20mm	2.17 ± 1.67mm	1.55mm (1.05 , 2.05) p<0.001	0.79	
Scapular movement	Low-force (22.3 ± 3.2N)	55.3 ± 0.6°	56.2 ± 1.1°	-0.9° (-1.63 , -0.21) p=0.015	1.02	F=19.649 p<0.001
	Medium-force (71.8 ± 8.7N)	55.7 ± 0.9°	59.5 ± 2.4°	-3.8° (-5.12 , -2.49) p<0.001	2.10	
	High-force (150.1 ± 22.1N)	56.8 ± 1.7°	65.3 ± 5.7°	-8.5° (-11.91 , -5.12) p<0.001	2.02	

GLDM, Glenohumeral lateral distraction mobilisation; N, Newtons; mm, millimeters; °, grades.

Table S3: Outcomes of the magnitude of force applied with GLDM, with and without scapular fixation in lateral movement of the humeral head and scapular movement, by dominance.

Variable	Magnitude of GLDM Force	Scapular Fixation	Non-scapular Fixation	Mean difference (95%CI)	Effect Size	p-value
Dominant						
Lateral movement of the humeral head	Low-force (22.0 ± 3.9N)	0.98 ± 0.54mm	0.89 ± 1.01mm	0.09mm (-0.58 , 0.76) p=0.769	0.11	F=21.972 p<0.001
	Medium-force (67.4 ± 9.3N)	2.19 ± 1.00mm	1.93 ± 1.69mm	0.45mm (-0.47, 1.00) p=0.448	0.19	
	High-force (147.6 ± 19.1N)	4.26 ± 2.01mm	2.73 ± 2.19mm	1.53mm (0.72 , 2.35) p=0.002	0.73	
Scapular movement	Low-force (22.0 ± 3.9N)	55.7 ± 1.3°	56.5 ± 1.4°	-0.8° (-1.57 , -0.02) p=0.046	0.59	F=37.906 p<0.001
	Medium-force (67.4 ± 9.3N)	56.6 ± 1.4°	60.2 ± 3.2°	-3.6° (-5.29 , -1.96) p=0.001	1.46	
	High-force (147.6 ± 19.1N)	58.2 ± 2.6°	65.9 ± 4.5°	-7.7° (-9.83 , -5.62) p<0.001	2.10	
Non-Dominant						
Lateral movement of the humeral head	Low-force (22.7 ± 3.9N)	1.03 ± 0.76mm	0.70 ± 0.53mm	0.33mm (-0.37 , 0.67) p=0.073	0.50	F=11.859 p<0.001
	Medium-force (68.5 ± 10.1N)	2.16 ± 1.29mm	1.37 ± 0.85mm	0.79mm (0.38 , 1.21) p=0.001	0.72	
	High-force (139.1 ± 20.8N)	3.23 ± 1.84mm	1.99 ± 1.26mm	1.24mm (0.71 , 1.77) p<0.001	0.87	
Scapular movement	Low-force (22.7 ± 3.9N)	55.2 ± 0.4°	56.9 ± 1.9°	-1.7° (-2.77 , -0.67) p=0.004	1.24	F=35.189 p<0.001
	Medium-force (68.5 ± 10.1N)	55.9 ± 1.1°	61.2 ± 3.7°	-5.3° (-7.18 , -3.44) p<0.001	1.94	
	High-force (139.1 ± 20.8N)	57.3 ± 2.2°	69.4 ± 7.1°	-12.1° (-15.88 , -8.37) p<0.001	2.30	

GLDM, Glenohumeral lateral distraction mobilisation; N, Newtons; mm, millimeters; °, grades.
